# Immune responses in the irritable bowel syndromes: time to consider the small intestine

**DOI:** 10.1186/s12916-022-02301-8

**Published:** 2022-03-31

**Authors:** Grace L. Burns, Nicholas J. Talley, Simon Keely

**Affiliations:** 1 NHMRC Centre of Research Excellence in Digestive Health, The University of Newcastle, Callaghan, New South Wales Australia; 2 College of Health, Medicine and Wellbeing, The University of Newcastle, Callaghan, New South Wales Australia; 3 Immune Health Program, Hunter Medical Research Institute, New Lambton Heights, New South Wales Australia

**Keywords:** Irritable bowel syndrome, Disorders of gut-brain interaction, Functional gastrointestinal disorders, Small intestine, Immune

## Abstract

**Background:**

Irritable bowel syndrome (IBS) is considered a disorder of gut-brain interaction (DGBI), presenting as chronic abdominal pain and altered defaecation. Symptoms are often food related. Much work in the field has focused on identifying physiological, immune and microbial abnormalities in the colon of patients; however, evidence of small intestinal immune activation and microbial imbalance has been reported in small studies. The significance of such findings has been largely underappreciated despite a growing body of work implicating small intestinal homeostatic imbalance in the pathogenesis of DGBIs.

**Main text:**

Small intestinal mechanosensation is a characteristic feature of IBS. Furthermore, altered small intestinal barrier functions have been demonstrated in IBS patients with the diarrhoea-predominant subtype. Small intestinal bacterial overgrowth and increased populations of small intestinal mast cells are frequently associated with IBS, implicating microbial imbalance and low-grade inflammation in the pathogenesis of IBS. Furthermore, reports of localised food hypersensitivity responses in IBS patients implicate the small intestine as the site of immune-microbial-food interactions.

**Conclusions:**

Given the association of IBS symptoms with food intake in a large proportion of patients and the emerging evidence of immune activation in these patients, the current literature suggests the pathogenesis of IBS is not limited to the colon but rather may involve dysfunction of the entire intestinal tract. It remains unclear if regional variation in IBS pathology explains the various symptom phenotypes and further work should consider the intestinal tract as a whole to answer this question.

## Background

Irritable bowel syndrome (IBS) is a disorder of gut-brain interaction (DGBI), the term for heterogeneous conditions of the gastrointestinal tract (GIT), previously described as ‘functional’ gastrointestinal disorders, for which there is no recognised overt structural pathology [1]. Instead, IBS is diagnosed based on a specific symptom profile, including abdominal pain in conjunction with alterations in bowel habit. IBS patients are subtyped based on bowel habit profile, into diarrhoea (IBS-D), constipation (IBS-C), mixed (IBS-M) or indeterminate, and it is suggested that approximately 10–20% of all cases develop after an episode of acute gastroenteritis (post-infectious, PI-IBS) [2]. Recent findings of subtle, sub-clinical gastrointestinal inflammatory changes in these patients indicate a role for the immune system in driving symptom onset and chronicity [3]; however, the nature of immune activation and involvement remains unknown. The absence of obvious pathology or a known trigger of immune involvement limits the therapeutic options and diagnostic approaches for these patients to often ineffective management of symptoms rather than specific treatment of the cause. It is hypothesised that IBS represents several conditions that result from dysfunction of the pathways that regulate homeostasis [4]; however, this is yet to be conclusively proven.

To date, most studies investigating the immune abnormalities in IBS have focused on the colon, given the association of the condition with altered defaecation patterns and technical difficulties in sampling biopsies or fluid from the small intestine (SI), particularly in larger studies. However, the SI is the major site of both nutrient antigen exposure and maintenance of immune tolerance against food and commensal microbes [5]. Combining these facts with emerging evidence for food intolerance as a driver of IBS [6, 7], the SI is increasingly being explored as a site of IBS pathology [8–10]. As such, we review the literature for SI involvement in immune responses in IBS and advocate for a more universal approach to examining immune activation across the diverse geography of the gut in future studies.

## Main text

### Regional specificity and homeostasis along the gastrointestinal tract

**Fig. 1:**
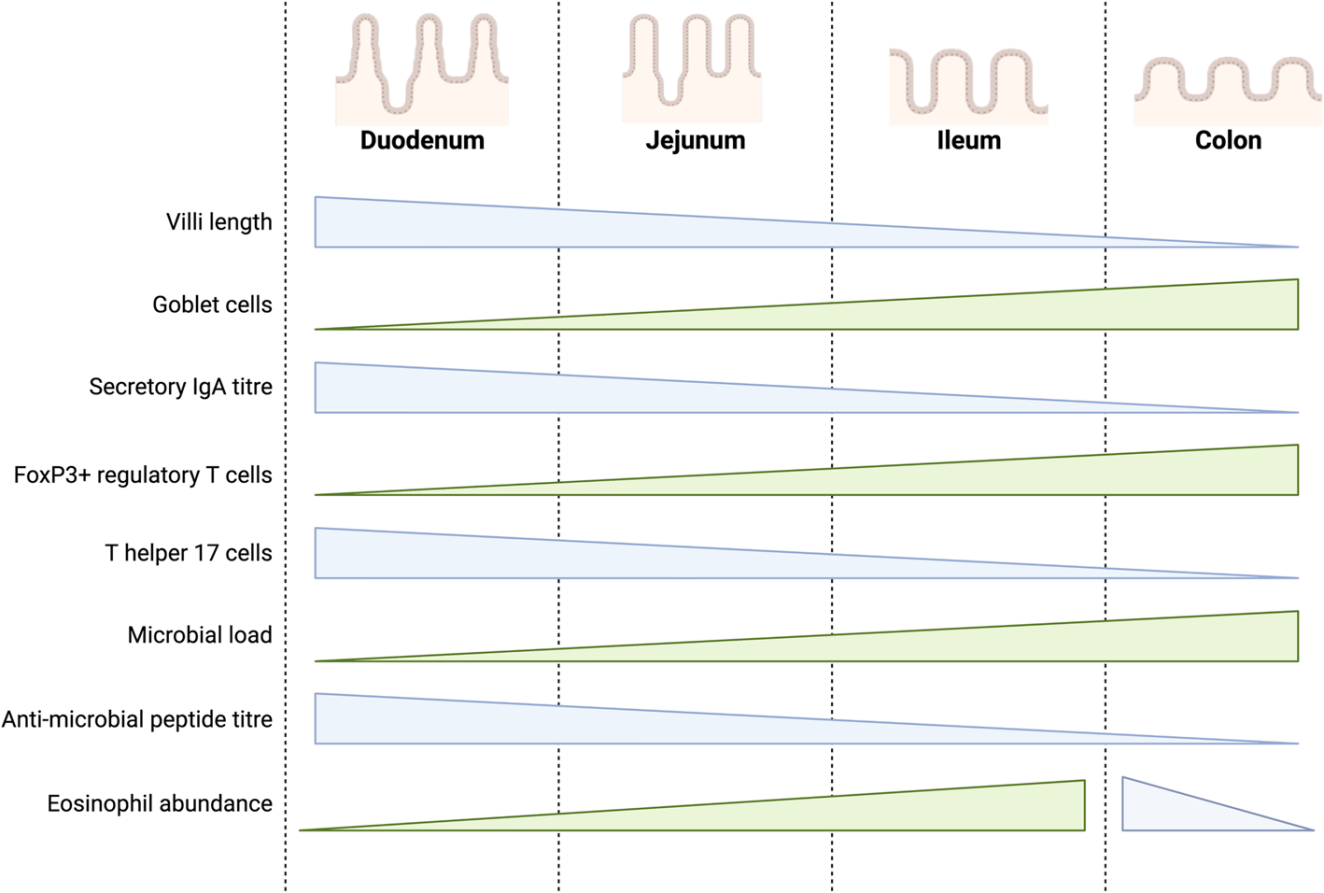
Regional specificity of selected immune and microbial components in the gastrointestinal tract. There is a distinct variation in the abundance of immune and microbial factors that mediate homeostasis in conjunction with physiological function throughout the small intestine and colon. Because of the role of the small intestine in nutrient absorption, the proximal segments (duodenum and jejunum) have longer, finger-like villi to increase the available surface area. The distal small intestine, the ileum, has shorter villi. Functionally, the colon primarily reabsorbs water and processes unabsorbable waste as faeces for elimination and does not have the finger-like projections of the small intestine. In the colon, immune homeostasis is primarily focused on tolerating the high commensal burden. As such, there is a higher abundance of Th type 17 cells in the duodenum that decreases towards the distal colon, corresponding with an inverse abundance of FoxP3^+^ regulatory T cells. Nutrient absorption capacity is greatest in the duodenum and decreases towards the colon. This corresponds with the small intestinal immune systems focus on oral tolerance to food antigens and production of anti-microbial peptide production and secretory IgA. Eosinophils are a normal constituent of the gastrointestinal tract. Their abundance increases towards the distal small intestine, peaking in the terminal ileum and proximal colon before decreasing towards the rectum [15–18]. The image was created using BioRender.com

The GIT exhibits regional specificity, best demonstrated by differences in the structure and function of the SI and colon. The primary function of the SI is the absorption of nutrients, with most absorption occurring in the duodenum and jejunum due to the increased surface area provided by the villi structures characteristic of these sites [11]. Colonic function, in contrast, is associated with the absorption of water and processing indigestible food material into faeces for elimination [11]. These site-specific functions are associated with physiological changes throughout the length of the GIT, with the SI exhibiting a lower pH [12] and shorter transit time than the colon [13]. In addition, there are site-specific selective pressures on the microbiota due to such physiological characteristics of the SI and colon [14]. Consequently, the immune profiles of the proximal and distal GIT are associated with geographical luminal signals (Fig. 1), such as exposure to dietary antigens in the SI and microbial signals in the colon [15], although much of this work has been demonstrated in animal models, rather than in humans.

In the SI, the immune system modulates homeostasis against luminal antigens by an active process known as oral tolerance [19] in conjunction with the small intestinal mucus layer, which facilitates closer contact of commensals with epithelial cells for sampling by antigen-presenting cells [20]. The discrimination of detrimental antigens from innocuous food proteins and commensals within the GIT is a complex process mediated by the actions of either reactive lymphocytes or regulatory T cells (Treg). Oral tolerance exists in order to prevent redundant and excessive immune responses to common food antigens and intestinal flora [21]. Ingested food proteins undergo a rigorous digestive process before reaching the small intestine, including digestion by proteases in the saliva, stomach and pancreatic acids [22]. Despite this digestive process, some proteins retain intact epitope structures that may come into contact with the mucosa of the lumen [23]. It is at this point that the immune system directs the development of tolerance against the specific epitope encountered to prevent unnecessary immune responses; however, in situations of homeostatic interruption, tolerance may be replaced by the induction of adaptive hypersensitivity immune responses.

### Involvement of the colonic adaptive immune system in IBS

While there is little consensus regarding the specific immune profile of IBS in the literature, largely due to the innate heterogeneity in the condition itself and methodological differences across studies [4], IBS patients seem to have greater basal levels of immune activation compared to outpatient or healthy control populations. A meta-analysis of cytokine studies [24] found an imbalance in the ratio of pro-inflammatory tumour necrosis factor (TNF) to interleukin (IL-)10. IL-10 is considered anti-inflammatory due to its capacity to limit T cell differentiation to prevent T helper (Th) cell polarisation [25], highlighting homeostatic imbalance as a feature of IBS. A small study of peripheral monocytes stimulated with lipopolysaccharide suggested monocytes from IBS patients were more mature [26] and IBS patients have higher levels of T cells expressing markers of activation compared to controls [27, 28]. Findings of altered Toll-like receptor (TLR) expression in the colon [29, 30] and elevated faecal antimicrobial β-defensin 2 levels [31] suggest activation of the innate immune system by microbial components may also contribute to disease pathogenesis, and this is supported by an exaggerated release of inflammatory cytokines (IL-1β, IL-6, IL-8 and TNF) from whole blood samples stimulated with TLR agonists in patients compared to healthy controls [32]. Importantly a meta-analysis of colonic immune cells highlighted regional and subtype-specific differences in immune cell numbers [33], supporting the notion of IBS as a condition not localised to one region of the colon.

The most consistently reported feature of IBS is increased mast cell numbers in both the SI and colon [34–40]. While some studies were unable to demonstrate altered mast cell numbers in the colon [41] likely due to methodological differences, sample sizes or selection bias, increased secretion of colonic tryptase and histamine [41–43] support a role for mast cells in IBS. Furthermore, the proximity of mast cells to enteric nerves correlates with the severity of abdominal pain, visceral hypersensitivity, fatigue and co-morbid depression [37, 44, 45] in IBS, suggesting a prominent role for these cells in both the pathophysiology and psychological burden of IBS. Similarly, colonic eosinophils have been reported as increased in patients [46–49]; however, this finding is not reproduced in all studies [50]. While there is a paucity of IBS studies examining the eosinophil number and activation status in the small intestine, one study found no change in duodenal eosinophil number in IBS patients compared to controls [35]. Eosinophils have gained prominence as an effector cell in other DGBIs including functional dyspepsia (FD), and given their relationship with mast cells in sensitisation-like immune responses [51], it is likely there is a functional role for eosinophils in SI immune activation in IBS. However, currently, the signals recruiting and activating these cells are unknown.

Given the finding of increased mast cells in IBS, a prominent hypothesis for immune activation is the notion of antigens, likely of food and/or microbial origins, stimulating the induction of a Th type 2 response. In this setting, antigens are presented to naïve T cells by antigen-presenting cells (such as dendritic cells) which drive differentiation into activated Th2 cells to stimulate immunoglobulin (Ig) E production from B cells. The subsequent binding of IgE to mast cells and re-exposure to antigen then results in degranulation and release of inflammatory mediators in close proximity to nerve cells that result in the onset of symptoms [52]. While one study demonstrated that stimulation of peripheral T cells from IBS patients resulted in increased production of IL-5 and IL-13 [53], a systematic review of the literature revealed there was little specific evidence for activation of this pathway in IBS [4].

The potential for Th17 responses in the microinflammatory profile of IBS has also been proposed, based on indirect evidence of increased peripheral TNF and IL-6 in patients [3, 54]. Th17 cells exist in a balance with Tregs to maintain gut immune homeostasis [55]; however, Th17 responses can also induce inflammation and autoimmune responses. For instance, in asthma, activation of Th17 pathways results in the release of IL-17, a cytokine which acts on the epithelium to drive recruitment of effector cells, including macrophages and eosinophils [56]. Interestingly, one study showed serum levels of IL-17a and TNF were significantly increased in conjunction with decreased IL-10 levels in patients with IBS-D [57], implicating an altered Th17/Treg axis in this subtype. While a meta-analysis of colonic immune cells in IBS found the total lymphocyte population (CD3^+^) was increased in patients, likely due to increased CD4^+^ cells [33], there are few studies examining the intestinal or colonic T cell phenotypes to support the Th2/Th17 hypotheses. Given both Th2 and Th17 immune responses would likely be occurring in relation to luminal antigens and the duodenum is where antigens initially interact with the immune system, the notion of IBS as a condition exclusively affecting the colon does not make sense. Rather, it is likely that IBS represents an adaptive immune response to luminal antigens that manifests heterogeneously along both the SI and colon.

### Evidence for small intestinal immune involvement in IBS

Reduced integrity of the mucosal barrier in IBS likely facilitates translocation of luminal antigens for direct contact with the immune system [58], which cyclically promotes continued permeability of the barrier. Increased SI [59, 60] and colonic barrier permeability have been associated with visceral hypersensitivity [59], independent of disease subtype [61], suggesting that a loss of barrier integrity may be the first step to priming of the immune system in IBS. However, one study identified SI permeability was attributable to the IBS-D subtype only, finding that altered SI permeability in IBS-C compared to controls was influenced by confounding lifestyle factors and that colonic permeability was unchanged when measured using multi-sugar testing [62]. Interestingly, dysregulated stress responses may mediate immune activation in the SI in IBS, given the association between corticotropin-releasing factor and jejunal mast cells and eosinophils [63, 64]. Such dysregulation has also been demonstrated in FD [65], and it remains to be seen if this pathway occurs in conjunction with or independently of the classical Th2-mediated response initially proposed to drive immune activation in IBS.

While the specific contribution of T cell populations to the presentation of IBS remains unclear, studies have suggested that IBS patients have a greater lymphocyte burden in the duodenum [35, 66] and jejunum [67], and there is no change in the total lymphocyte density in the ileum [45, 68]. Early work using the first iteration of the Rome criteria [67] identified subclinical increases in intraepithelial lymphocytes (IEL) and infiltration of lymphocytes into the jejunal myenteric plexus in IBS patients when compared to outpatient controls. The increase in IEL number has been reported in further studies, specifically in IBS-D [36, 66] and in the terminal ileum [69], and suggests enhanced surveillance of luminal content or lingering hyperreactivity of the SI immune system. Given the role of the myenteric plexus in the coordination of contraction and motility [70], lymphocytic infiltration at this site suggests subclinical inflammation specific to the enteric system that may be linked to motility dysfunction in patients. In addition, qualitative assessment of jejunal mast cells found no difference between patients and controls [67], a finding that was later supported by a systematic review and meta-analysis of mast cells in the SI [40], which identified increases in ileal, but not duodenal or jejunal mast cells in IBS patients. One of the only population studies to examine SI pathology in IBS identified increased IELs (specific to IBS-C only) and mast cells in the duodenum of both diarrhoea and constipation subtypes [35]. While the literature is conflicting regarding the role of lamina propria lymphocytes in the SI in IBS, likely due to variation in methodology for quantification and patient categorisation, it does appear that alteration in immune cell populations is a SI feature of a subset of patients. Interestingly, increased lamina propria lymphocyte populations were not reported in the ileum in two studies [45, 68], while the meta-analysis [40] suggested that increased SI mast cells are a feature specifically of the ileum. Unfortunately, progress towards confirming and understanding an immune mechanism to drive symptom chronicity in IBS will depend on the identification of specific antigenic triggers, and given the heterogeneity among patients, this process will be complicated given it is likely that no single antigen is responsible for IBS symptoms.

### Food antigens as a trigger for IBS symptom onset

Up to 84% of IBS patients self-report that food ingestion induces symptoms, with incompletely absorbed carbohydrate sources (such as dairy, beans and some fruits) and foods that drive histamine release (including milk, beer and pork) most reported with symptoms [71]. Furthermore, the exclusion of foods with raised IgG titres in a trial of 150 patients resulted in a significant symptom reduction at 12 weeks [72], linking food-driven responses to symptom burden. The introduction of specific foods directly to the duodenum of IBS patients using confocal laser endomicroscopy (CLE) showed that 70% of these patients had a detectable response (CLE+) to one or more foods [7]. Furthermore, there were significant differences in the immune activation profile of CLE+ and CLE− IBS patients, characterised by increases in IEL counts, increased claudin-2 and decreased occuludin levels, suggestive of barrier dysfunction in CLE+ patients. This response profile was not associated with systemic IgE; however, CLE+ IBS patients had higher levels of eosinophil degranulation [7], suggestive of a non-IgE-mediated food intolerance. The findings of this study confirm the capacity for a SI immune response to food in a subset of susceptible IBS patients. However, it is unclear how prevalent this phenomenon is in IBS patients, and there are likely other mechanisms by which IBS symptoms may manifest. Such differences in manifestations of IBS would help to explain the heterogeneity reported regarding immune activation profiles in the literature [4]. A more recent study demonstrated localised responses to injection of food antigens in the recto-sigmoid region of IBS patients, characterised by oedema, IgE antibody production and mast cell activation at the challenge site [6]. While intact food proteins are unlikely to make direct contact with the rectosigmoid mucosa during the process of digestion, this study demonstrates immune responses to food are localised to the intestinal mucosa in IBS. Here, loss of oral tolerance to common food antigens may result from heightened immunosurveillance and drive visceral hypersensitivity [6]. When considered with studies using CLE to examine responses to antigen [7], these findings would suggest that both classical (IgE-mediated) and non-classical hypersensitivity pathways may be activated in IBS patients. Importantly, the identification of these localised immune responses with no systemic profile suggests the need for caution when interpreting studies of systemic mediators (e.g. serum cytokines or peripheral blood mononuclear cell populations) in IBS patients.

### Small intestinal bacterial overgrowth (SIBO) and the microbiota in IBS

The colonic and faecal microbiomes have been profiled in IBS patients and suggest altered composition is a feature of IBS [73–76]. The findings of a systematic review suggested specific phylums (Proteobacteria and Bacteroidetes) and families (Enterobacteriaceae and Lactobacillaceae) of bacteria likely contributed to a pro-inflammatory microenvironment in the colon in IBS [73]. Furthermore, a systematic review of faecal microbiota transplantation (FMT) for IBS found that administration of the transplant to the small intestine was effective, and the placebo effect for this route of administration was lower compared to FMT administered via colonoscopy [77]. Such data suggests targeting of the SI microbiota may be an effective approach to treating IBS, and findings of altered anti-microbial defence factors (such as TLRs and β-defensin 2, as previously discussed) in patients highlight dysregulation of the immune response to commensals may be associated with immune activation. Increased production of IgA in the terminal ileum may be a consequence of shifts in the total microbial composition [78], resulting in local inflammatory signals. However, the lack of consistent microbial profiling methods and patient characterisation combined with a paucity of functional data regarding the altered microbes limits our understanding of the specific species and taxa that may contribute to IBS. Furthermore, there are very little data regarding the SI luminal or mucosa-associated microbiome outside of the context of SIBO.

SIBO describes excessive overgrowth of colonic-type bacteria, classically defined as > 10^5^ colony-forming units per millilitre of upper gastrointestinal aspirate [79] although a cut-off of 10^3^ has been suggested more recently [80]. SIBO has been associated with IBS, although the literature is conflicting regarding whether the association is specific to one subtype over another [81–83]. The frequency of SIBO in IBS patients has been reported in the range of 4–78% [84]; however, there is substantial variation in the methodologies used to quantify bacterial load and a lack of consensus diagnostic criteria that have hampered efforts to firmly investigate the relationship between SIBO and IBS to date [85]. While breath testing has become the preferred diagnostic choice given the non-invasiveness and simplicity of such approaches [86], it is of note this method is influenced by gut transit and patient factors including physical activity [87] and pausing medications such as proton pump inhibitors before the test [88] and has poor correlation with gold standard aspirate cultures [89]. As such, it is difficult to determine if the abnormal test results reflect the presence of SIBO, or more rapid transit and fermentation by colonic bacteria [89]. There is a suggestion that the expansion of a colonic-like microbial profile in the small intestine triggers the immune system to induce the low-grade inflammatory state and induces hypersensitivity responses. In this scenario, the overgrowth of particular species stimulates immune cells to secrete pro-inflammatory cytokines, including IL-1α and IL-1β [90], that drive recruitment of effector cells, such as mast cells, and impair the mucosal barrier. Furthermore, treatment with oral rifaximin, a broad-spectrum antibiotic that acts in the SI, has been shown to relieve symptoms of abdominal bloating and pain in IBS-D patients [91]. Animal studies suggest the efficacy of rifaximin may be due to its capacity to downregulate inflammatory cytokines including IL-17, IL-6 and TNF while improving intestinal barrier permeability and reducing visceral hypersensitivity [92]. These findings highlight that not only is the SI involved in IBS but targeting of microinflammation at this site may result in improved symptom burden.

**Fig. 2:**
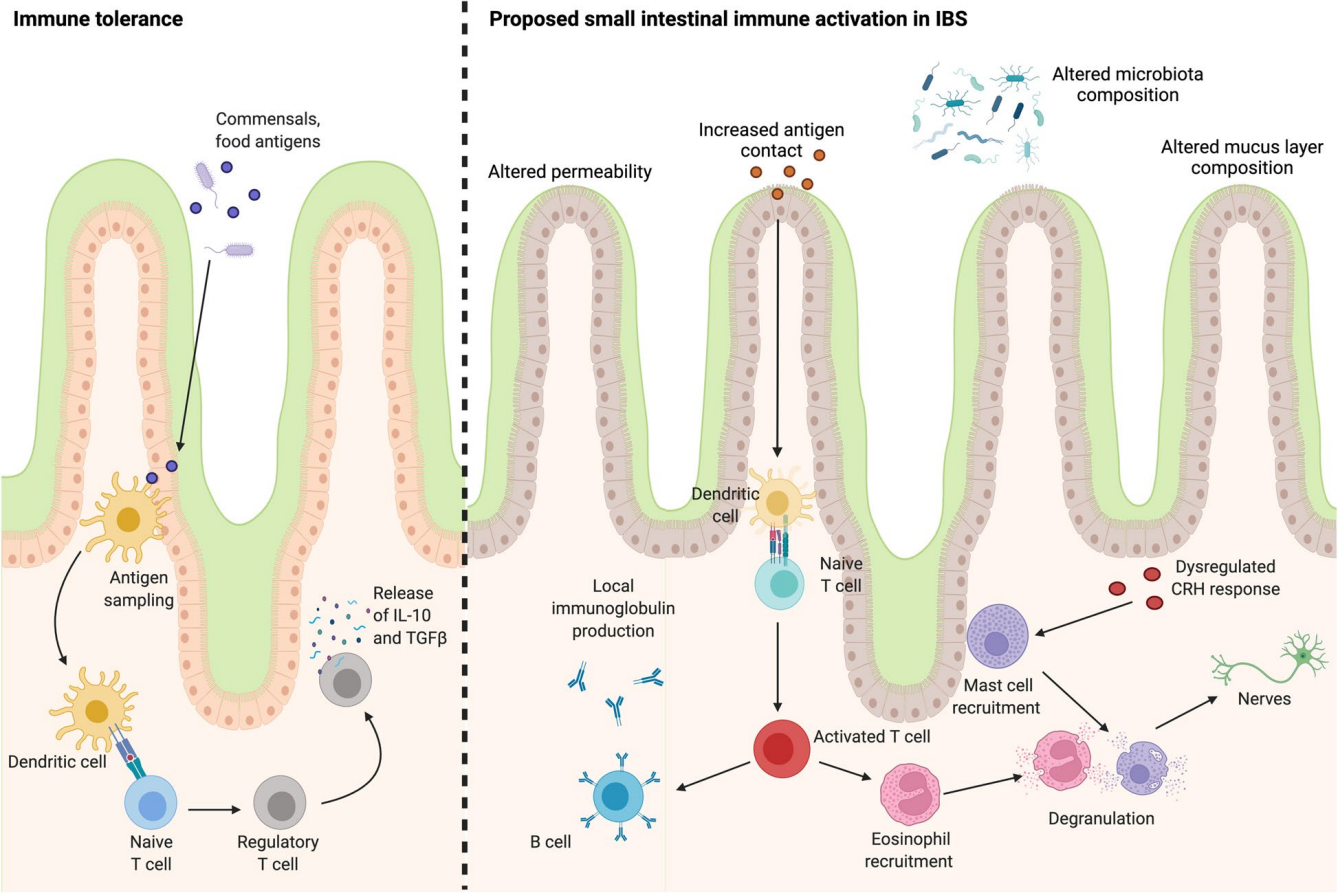
Hypothesised immune mechanisms potentially involved in small intestinal dysfunction in IBS. The small intestinal immune system actively modulates tolerance to commensal microbes and food components to maintain homeostasis, in conjunction with the mucosal barrier and mucus layer. In this process, antigens are sampled by dendritic cells and presented to naïve T cells. The differentiation of these cells to regulatory T cells results in the release of interleukin-10 and transforming growth factor beta, which actively suppresses inflammatory immune responses. In contrast, physiological abnormalities in the composition of the mucus layer, coupled with altered mucosal permeability and changed microbial community composition in IBS patients may allow for increased antigen contact with the mucosa and a dysregulated or increased stress response. In this environment, antigen presentation may result in the activation of T cell subsets that drive B cell maturation and specific antibody production that is likely localised to the gastrointestinal tract. The activation of the adaptive immune system may drive the recruitment of eosinophils and mast cells, which degranulate and release inflammatory mediators. The release of these mediators near enteric nerves is likely to promote abnormal signalling and may result in visceral pain. Altered or enhanced stress signalling may also enhance this eosinophil and mast cell response to further contribute to immune activation. However, these pathways require further investigation in IBS patients compared to controls to demonstrate the mechanisms underlying intestinal immune activation. The image was created using BioRender.com

Significant progress has been made in characterising IBS as a condition of disordered interactions between the gut and the brain with microinflammation as a central pathology. This concept has directly challenged the notion of this condition as a ‘functional’ disorder, given the significant array of physiological, microbial and immune abnormalities described. However, it is time to progress the field further towards greater consideration of the role of the SI in this condition. While this is not a new concept, given early reports of altered SI permeability in IBS patients, the SI has largely been ignored in the search for targetable mechanisms that underlie GI dysfunction in IBS. The literature suggests that homeostatic imbalance is not limited to the colon, given that the immune system, microbiota and physiological function of the SI are affected by IBS. A proposed pathway for small intestinal immune activation in IBS is included in Fig. 2. What is unclear is if the involvement of the SI is a feature of a subset of patients, common to all or is instead a previously unrecognised link between IBS and other DGBIs, such as functional dyspepsia. FD affects the gastroduodenal region, with altered barrier function and microinflammation described in the duodenum [93]. Like IBS, food and microbial antigens are hypothesised as responsible for cyclic episodes of symptom onset due to subclinical inflammation. Interestingly, studies suggest between 26.7 and 48.7% of IBS patients meet the Rome criteria for concurrent FD [94]. In addition, this patient subset reports greater symptom severity and decreased quality of life compared to patients with only one DGBI [95], leading to questions of whether these are distinct conditions or rather different manifestations of the same process of homeostatic imbalance and microinflammation. In support of this, one hypothesis suggests that the site of gastroenteritis predicts the development of post-infectious DBGIs [96, 97], whereby infections in the proximal SI are more likely to result in FD development, while distal infections may predispose to onset of IBS and if both regions are involved, then overlapping FD/IBS may develop. However, currently, there is little prospective data to support this concept.

While the literature regarding the phenotype of T cell activation in IBS is underdeveloped, it is worth considering that significant alterations in individual effector T cell populations are unlikely in IBS. Rather, future studies should consider the deep characterisation of the effector and memory T cell populations in both the SI and colon. If these are in fact disorders of homeostatic imbalance, it is likely that the T cell repertoire is instead characterised by shifts in the balance of regulatory, effector and memory T cells.

## Conclusions

Despite reports of SI alterations in physiology, microbial communities and immune activation in IBS patients, many studies continue to focus solely on the colon. However, dysfunction of the entire intestinal tract may be implicated in IBS, and this will be an important consideration in future studies as we move towards identifying specific triggers and immune pathways that drive symptom chronicity. Characterisation of both SI and colonic immune profiles in large cohorts will be critical to unravelling the heterogeneity inherent to IBS and may eventually identify distinct subgroups of people based on responses to food and/microbial luminal antigens, allowing for specific therapeutic targeting.

## Data Availability

N/A.

## Abbreviations

CD: Cluster of differentiation
CLE: Confocal laser endomicroscopy
DGBI: Disorder of gut-brain interaction
FD: Functional dyspepsia
GIT: Gastrointestinal tract
IBS: Irritable bowel syndrome
IBS-C: Irritable bowel syndrome constipation
IBS-D: Irritable bowel syndrome diarrhoea
IBS-M: Irritable bowel syndrome mixed
IEL: Intraepithelial lymphocytes
Ig: Immunoglobulin
IL: Interleukin
PI-IBS: Post-infectious irritable bowel syndrome
SI: Small intestine
SIBO: Small intestinal bacterial overgrowth
Th: T helper
TLR: Toll-like receptor
TNF: Tumour necrosis factor
Treg: Regulatory T cells
